# Mild generalised hypermobility (Beighton score ≥2) is the most accurate preoperative indicator of intraoperatively‐confirmed microinstability in 622 primary hip arthroscopies

**DOI:** 10.1002/jeo2.70955

**Published:** 2026-10-21

**Authors:** Catherine J. Bacon, Rowan P. Auchterlonie, Rebecca M. Woodward, Gen Lin Foo, Man Lu, Renuka M. Vesey, Matthew J. Brick

**Affiliations:** ^1^ Orthosports North Harbour Ltd., Millennium Centre Auckland New Zealand; ^2^ School of Nursing, Waipapa Taumata Rau/University of Auckland Auckland New Zealand; ^3^ Te Whatu Ora/Health New Zealand Auckland New Zealand; ^4^ Faculty of Medical and Health Sciences, Waipapa Taumata Rau/University of Auckland Auckland New Zealand; ^5^ Auckland Radiology Group Auckland New Zealand; ^6^ Apex Sports Clinic Singapore Singapore

**Keywords:** arthroscopic surgery, capsule, hip joint, instability, laxity

## Abstract

**Purpose:**

To identify the preoperative diagnostic accuracy of routine hip arthroscopy clinical assessments for intraoperatively confirmed microinstability.

**Methods:**

Image indices, clinical assessments and operation details from 622 consecutive primary hip arthroscopies undertaken by a single surgeon between January 2018 and December 2020 were extracted from the surgeon's database. Microinstability diagnosis was retrospectively confirmed by the presence of any of four intraoperative criteria: increased ease of distraction of the hip, isolated straight anterior labral tear, inside‐out chondral lesion or a lateral Seldes 2 or 3 labral tear in the absence of cam or pincer morphology. Optimal thresholds for numerical measures were determined using receiver operating characteristic curve (ROC) analyses by minimising the distance from the point of perfect sensitivity and specificity. Diagnostic parameters, calculated from contingency tables, were evaluated for individual and combinations of demographic, examination and imaging indices, including age, self‐reported gender, risk‐sport participation, positive log roll/hip dial test, Beighton score, hip range‐of‐motion, lateral centre‐edge angle (LCEA), Tonnis angle, crossover sign and ischial sign.

**Results:**

A total of 622 hips (533 patients) aged 35.5 ± 11.8 years, 56%:44% female:male ratio were identified. Female patients were 2.08 times more likely than male to have microinstability (sensitivity 0.93, specificity 0.55). A Beighton score ≥2 was the best overall indicator of microinstability (sensitivity 0.85, specificity 0.82, positive likelihood ratio 4.73, negative likelihood ratio 0.18). Presence of female gender, with either LCEA < 35° or Beighton score ≥2, increased sensitivity to 0.93, at the expense of specificity (0.69) and no combination of indices surpassed Beighton score alone in the Youden index (sensitivity + specificity − 1).

**Conclusion:**

Among the individual and combined presurgical indicators evaluated, Beighton score ≥2 was the most accurate test for intraoperatively‐confirmed microinstability in primary hip arthroscopy patients. Mild generalised laxity, particularly in female patients with a slightly shallow acetabulum (LCEA < 35°), should raise suspicion of microinstability to minimise missed diagnoses.

**Level of Evidence:**

Level III, diagnostic study.

**Abbreviations**
AB: HEER abduction‐hyperextension‐external rotation
FAI: femoroacetabular impingement
HEER: hyperextension‐external rotation
LCEA: lateral centre‐edge angle
ROC: receiver operating characteristic

## INTRODUCTION

Hip joint instability, resulting from abnormal translational movement of the femoral head within the acetabulum, may contribute to hip pain and symptoms for some patients presenting for hip arthroscopy surgery [8, 21]. Microinstability refers to atraumatic symptomatic hip instability that results from combinations of subtle skeletal abnormalities, such as abnormal acetabular or femoral version and acetabular dysplasia, or soft tissue problems, such as capsular laxity or injury and muscular weakness [21]. Repetitive rotational loading of the hip or extreme ranges of motion can contribute [42]. Although there is some dispute whether ‘microinstability’ is distinct from hip instability [38], the term ‘microinstability’ has been used throughout this article.

Accurate diagnosis of microinstability, a rapidly evolving clinical concept, can inform optimal arthroscopic management, particularly with respect to capsular management and appropriate osteochondroplasty to avoid iatrogenic instability. An extensive range of indicators of microinstability have been proposed [10, 24, 51]. These may be identified from patient history, clinical examination, imaging or surgical findings, and can be related to iatrogenic complications that compromise bony and/or capsular stability following correction of femoroacetabular impingement (FAI)‐related morphology [10, 50]. A 34‐item diagnostic tool developed through Delphi consensus by a large panel of orthopaedic surgeons and physical/physio‐therapists has been mooted as a first step towards standardising a clinical diagnosis [24]. Despite the myriad of proposed indicators, their relative importance remains uncertain, and there is limited information about their diagnostic accuracy.

A recent consensus paper has identified eight intraoperative findings that aid in microinstability diagnosis from 17 considered signs [43]. Confirmatory arthroscopic diagnosis of microinstability is considered the ‘gold standard’, and diagnostic markers can be broadly categorised as relating to either ease of distraction, pattern or location of chondrolabral damage, or capsular lesions [43]. Although a few studies have identified characteristics associated with intraoperatively confirmed microinstability, most, with some exceptions [35, 36], are in small cohorts of around 30 or fewer patients [1, 6, 22, 27].

The aim of this study was to determine the prevalence and diagnostic accuracy of preoperative characteristics associated with microinstability, confirmed intraoperatively from a retrospective analysis of consecutive primary hip arthroscopies performed by a single high‐volume surgeon. We approached this analysis without a hypothesis about which indicators would display the greatest diagnostic accuracy, though we expected that the thresholds would be similar to those suggested by previous consensus [24].

## METHODS

Arthroscopies included in this retrospective analysis were systematically identified from the surgeon's database. These operations were performed for the treatment of chondrolabral injury, normally arising from FAI with or without acute trauma, as previously described [4, 9, 17, 37]. Patients provided informed consent for the collection and research use of their information, and the data acquisition process has ethical approval as an observational study from the New Zealand Health and Disabilities Ethics Committees (17/NTA/269).

Consecutive primary hip arthroscopies performed by the surgeon between January 2018 and December 2020 were identified from the database. Procedures entailing only extraarticular endoscopy were excluded, as were those with previous ipsilateral extra‐ or intraarticular surgery involving the hip, groin or buttock region. Surgeries were classified as having cam, pincer or combined morphology of FAI syndrome based on clinical examination, performed and recorded by the surgeon, and imaging [18]. Image indices in the database are measured and recorded by the surgeon at the time received, although a small proportion (<5%) of missing indices were measured later by a researcher trained by the surgeon (M.L.). Arthroscopies in hips with a primary diagnosis of dysplasia (lateral centre‐edge angle [LCEA] < 18°), acute intraarticular fracture, dislocation or recent history of significant trauma were also excluded, as a diagnosis of microinstability could not be established for these cases.

Retrospective diagnosis of microinstability, based on operating notes and intraoperative images, was made by a researcher (R.P.A.), who was blinded to preoperative clinical history, examination and postoperative outcomes. Based on the available literature [43]. and the variables that were consistently available in our database, a positive diagnosis was indicated by any of the following criteria: increased ease of distraction of the hip, an isolated straight anterior labral tear, inside‐out chondral lesion or a lateral Seldes 2 or 3 labral tear in the absence of cam or pincer morphology.

Main sports and competitive levels were reported by patients in an online history questionnaire. Log roll/dial test [13, 40], Beighton screen [5] and range of hip motion: supine flexion and extension and seated external and internal hip rotation relative to neutral anatomical position were undertaken by the surgeon (M.J.B.) and recorded during presurgery clinical assessment. The presence of a crossover or ischial spine sign, LCEA and Tonnis/acetabular roof angle were assessed on antero‐posterior pelvis radiographs [30]. LCEA was the angle between a line at 90° to the transverse axis of the pelvis and a line from the centre of the femoral head to the lateral edge of the acetabular sourcil [31]. Based on previous literature [10, 24, 51], presurgery history, examination and imaging indicators suggestive of microinstability were extracted from the database. Those available included:

- self‐reported female gender [24];
- younger age;
- competitive or elite participation in sports demanding extreme flexibility or axial loading, such as gymnastics, dance/ballet, martial arts, figure skating, tennis, soccer, softball/baseball, golf or skating [24];
- generalised hypermobility reported or indicated by Beighton score [1, 24, 45];
- a positive log roll or hip dial test [7, 8, 21, 24];
- a high degree of internal or external hip rotational mobility in either hip [24];
- lower LCEA [20, 33, 47];
- a crossover sign or ischial spine sign indicated on radiograph [24];
- Tonnis angle >10° [24].

Statistical analysis was undertaken using IBM SPSS Statistics (v31; IBM Corporation). The distribution of continuous variables was assessed for normality via visual inspection (for *n* > 200), or for skewness or kurtosis falling outside the 99% confidence interval (*n* = 50–200) or 95% confidence interval (*n* < 50) [16]. Differences between groups were analysed using independent *t*‐tests for continuous variables and chi‐squared analysis for categorical variables. Each indicator of possible microinstability was analysed for its accuracy in detecting intraoperatively diagnosed microinstability. For numerical variables (ordinal and scalar), receiver operating characteristic (ROC) curves were used to determine statistically optimal thresholds for detecting microinstability. The threshold showing the greatest sum of sensitivity and specificity (and Youden Index = sensitivity + specificity – 1 was applied [2]. Specificity, sensitivity and likelihood ratios were determined for optimal and previously suggested thresholds for the detection of intraoperatively‐confirmed microinstability.

## RESULTS

From a total of 725 consecutive hip arthroscopies within the date range, 101 were excluded as secondary (revision or reoperative) procedures, 1 with severe acute traumatic injuries and 1 with an excision of heterotropic ossification; leaving a total of 622 primary arthroscopies (Figure 1).

**Figure 1:**
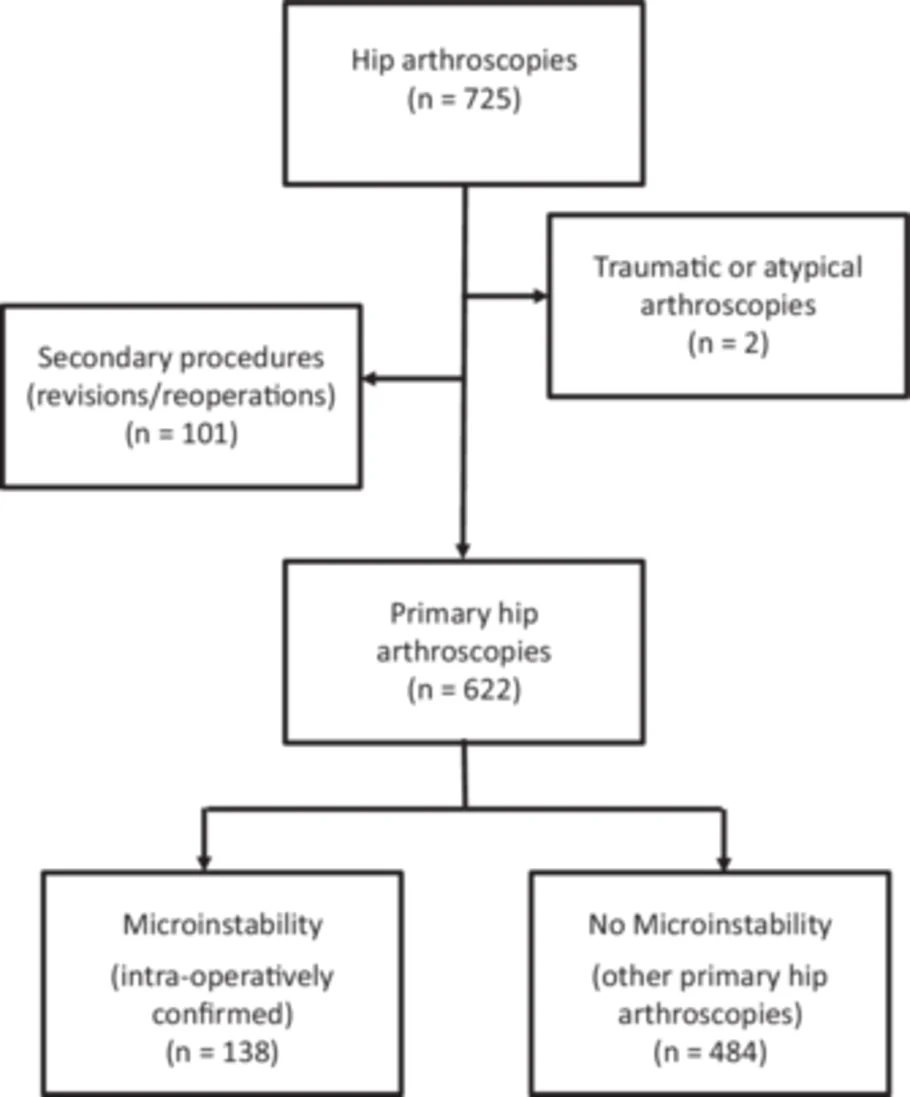
Selection of surgeries undertaken within the date range.

The 622 arthroscopies were performed on 553 different patients, mean age 35% and 56% female, primarily for FAI with either cam or pincer morphology or a combination of these (Table 1). Only 52 hips (8.4%) presented with microinstability in isolation (without cam or pincer morphology) (Table 1).

**Table 1:** Broad classification of primary diagnosis and hip arthroscopy procedure.

Main procedure	Number	Capsular plication^f^	Gender: female:male	Age (years)	BMI (kg/m^2^)
Cam^a^	445 (71.5)	91 (20.4)	222:223 (49.9:50.1)	35.1 ± 11.6	25.4 ± 4.2
Pincer^b^	18 (2.9)	3 (16.7)	17:1 (94.4:5.6)	39.2 ± 11.9	25.7 ± 4.5
Combined^c^	96 (15.4)	5 (5.2)	45:51 (46.9:53.1)	38.5 ± 11.5	26.0 ± 4.7
Microinstability^d^	52 (8.4)	52 (100)	52:0 (100:0)	30.2 ± 9.8	24.4 ± 6.0
Chondrolabral repair^e^	11 (1.8)	0 (0)	10:1 (90.9:9.1)	42.3 ± 16.9	23.3 ± 4.0
Total	622	151 (24.3)	346:276 (55.6:44.4)	35.5 ± 11.8	25.4 ± 4.5

*Note*: Data are number (percent) of procedures for frequencies or mean ± standard deviation (SD) for age and body mass index (BMI).

^a^ A cam diagnosis was applied when any femoroplasty was undertaken, including minor femoral head‐neck contouring for small cam.

^b^ A pincer diagnosis was applied when acetabuloplasty was performed and lateral centre‐edge angle (LCEA) was ≥40° or there was a diagnosis of focal anterior overcoverage.

^c^ A combined diagnosis was applied when the criteria for both cam and pincer were met.

^d^ Microinstability or chondrolabral repair identified in isolation without any diagnosis or treatment for cam or pincer morphology.

^e^ A chondrolabral repair surgeries included repair to chondrolabral soft tissue without osteoplasty.

^f^ Number receiving capsular plication (percentage of those in category) for suspected joint instability (microinstability).

Of the 622 arthroscopies, 138 (22%) had intraoperative confirmation of microinstability. Of approximately a third (34.8%) with at least one microinstability indication (Table 2), only 126 (53%) had intraoperatively confirmed microinstability (positive predictive value). Conversely, the negative predictive value of a single indicator was much higher at 96.9%: among those without any preoperative indicators, only 12 (3.1%) displayed intraoperatively confirmed microinstability. Of 14 continuous variables, 2 failed to meet assumptions of normality (flexion on the index and opposite hips), though deviations in skewness and kurtosis were just outside the specified limits. Parametric statistical tests were applied for all between‐group comparisons. Characteristics of those with and without an intraoperative diagnosis of microinstability are shown in Table 3. Notably, despite including those with LCEA ≥ 18°, the minimum observed was 21°.

**Table 2:** Preoperative indicators of hip microinstability in the primary hip arthroscopy cohort.

Criterion	Number (%)
Participant in risk sport^a^	12 (1.9)
Beighton score ≥4	133 (21.4)
Positive log roll/hip dial test	41 (6.6)
High degree of rotational mobility^b^	118 (19.0)
Borderline dysplasia (LCEA 21°–25°)	35 (5.6)
Crossover sign	46 (7.4)
Ischial spine sign	3 (0.5)
Tonnis angle >10°	4 (0.6)
At least 1 criterion	239 (34.8)

Abbreviation: LCEA, lateral centre‐edge angle.

^a^ Risk sports include gymnastics, dance/ballet, martial arts, figure skating, tennis, soccer/football, softball/baseball, golf or skiing; [24].

^b^ internal or external rotation >60° [24], or internal plus external rotation of ≥100°.

**Table 3:** Characteristics of the cohort according to intraoperatively confirmed hip microinstability.

	*n* available	Microinstability (*n* = 138)	Other primary hip arthroscopies (*n* = 484)	*p‐*Value^d^
		mean ± SD	mean ± SD	
Age at surgery (years)	622	32.1 ± 10.1	36.4 ± 12.1	<0.001
BMI (kg/m^2^)	616	24.7 ± 5.0	25.6 ± 4.3	0.04
LCEA (°)	618	32.1 ± 4.7	35.6 ± 6.1	<0.001
Alpha angle (°)	411	47.5 ± 8.6	56.6 ± 12.0	<0.001
Offset (mm)	473	7.3 ± 2.5	4.9 ± 3.2	<0.001
Tonnis angle (°)	40	6.5 ± 4.3	4.7 ± 4.7	0.29
Sup flexion (index)	162	98 ± 10	92 ± 08	<0.001
Sup flexion (opp)	156	101 ± 9	93 ± 7	<0.001
External rotation (index)	265	60 ± 16	49 ± 13	<0.001
Internal rotation (index)	266	34 ± 13	20 ± 14	<0.001
Ext + Int rotation (index)	263	95 ± 24	69 ± 23	<0.001
External rotation (opp)	258	64 ± 14	54 ± 12	<0.001
Internal rotation (opp)	259	39 ± 11	27 ± 14	<0.001
Ext + Int rotation (opp)	256	102 ± 20	81 ± 23	<0.001
		number (%^c^)	number (%^c^)	
Gender F:M	346:276	129:9 (93.5:6.5)	217:267 (44.8:55.2)	<0.001
Log roll/dial test (index)	60	25 (65.8)	14 (63.6)	0.87
Log roll/dial test (opp)	34	21 (84.0)	6 (66.7)	0.71
Beighton score	513			<0.001
0–3		33 (25.2)	347 (90.8)	
4–5		35 (26.7)	17 (4.5)	
6+		63 (48.1)	18 (4.7)	
Borderline dysplasia (LCEA 21°–25°)	618	14 (10.1)	21 (4.4)	0.01
Tonnis angle >10°	40	2 (18.2)	2 (6.9)	0.29
Crossover sign				
Large	530	0 (0.0)	6 (1.5)	0.09
Any	622	6 (4.3)	41 (8.5)	0.11
Ischial spine sign	622	1 (0.7)	2 (0.4)	0.64
Main sport level	517			0.25
No sport		14 (11.2)	34 (8.7)	
Occasional sport		32 (25.6)	74 (18.9)	
Frequent sport/training		54 (43.2)	179 (45.7)	
High level/professional		25 (20.0)	105 (26.8)	
Risk sport participant^a^	37	5 (62.5)	7 (24.1)	0.04
High‐risk sport participant^b^	37	4 (50.0)	1 (3.4)	<0.001

Abbreviations: BMI, body mass index; Ext, external; Int, internal; LCEA, lateral centre‐edge angle; Offset, femoral head‐neck offset; Opp, opposite hip; SD, standard deviation; Sup, hip movement completed in supine position.

^a^ Any of the following identified as main sport(s): gymnastics, dance/ballet, martial arts, figure skating, tennis, soccer/football, softball/baseball, golf, skiing.

^b^ Any of the following identified as main sport(s): gymnastics (including cheerleading/acrobatics), dance/ballet.

^c^ Percent of those available for analysis in each group.

^d^ *P* value for the chi‐squared test for categorical variables and independent t‐test for continuous variables with correction when Levene's test for equality of variances violated.

Although a Beighton score between 0 and 3 was recorded for 135 surgeries (55 scored 0, 32 scored 1, 30 scored 2 and 18 scored 3), a further 245 had Beighton recorded as ‘normal <4’. For analysis, these cases were assigned a numerical value of 1.5 (the mid‐point of the possible score range: 0–3). ROC analyses for the full cohort showed the Beighton score had the greatest area under the curve, with the optimal threshold for detecting intraoperatively‐confirmed microinstability being ≥2 for the Beighton score (Table 4). We repeated this analysis, recoding the 245 ‘normal’ Beighton scores first as 0 and then as 3, at either end of the normal range. Coding these as zero did not alter the optimal threshold for detection of microinstability (≥2); coding these as 3 changed the threshold to ≥4. Full range of hip rotation exhibited a greater area under the curve than either internal or external rotation alone, and optimal thresholds for hip external and internal rotation on the index side were 5° lower than on the opposite side, and 25° lower for internal rotation than for external rotation on both sides (Table 4).

**Table 4:** Receiver operating characteristic (ROC) analysis for scalar indices of intraoperatively‐confirmed microinstability.

	*n* (%) available	Optimal Threshold^a^	AuC^b^
Age (years)	622 (100)	<38.5	0.60 (0.55–0.65)
Beighton score	513 (82.5)	≥2	0.85 (0.80–0.90)
External rotation index (°)	265 (42.6)	≥55	0.73 (0.66–0.79)
Internal rotation index (°)	266 (42.8)	≥30	0.77 (0.72–0.83)
External+internal rotation index (°)	263 (42.3)	≥80	0.79 (0.74–0.85)
External rotation opposite (°)	258 (41.5)	≥60	0.71 (0.65–0.77)
Internal rotation opposite (°)	259 (41.6)	≥35	0.74 (0.68–0.80)
External+internal rotation opposite (°)	256 (41.2)	≥95	0.77 (0.71–0.83)
Lateral centre‐edge angle (°)	618 (99.4)	<35	0.67 (0.62–0.72)
Tonnis angle (°)	40 (6.4)	≥7	0.60 (0.40–0.80)

^a^ Optimal threshold is the average between two consecutively ordered values whose cut‐point defines the optimal detection of intraoperatively confirmed microinstability, according to the minimum value of the formula square‐root ([1‐sensitivity]^2^ + [1 – specificity]^2^).

^b^ Area under the curve (AuC) for the ROC analysis with 95% confidence interval in parentheses.

Sensitivity and specificity for the detection of intraoperatively diagnosed microinstability from individual indicators are shown in Table 5. Apart from some indicators expected to show very high specificity due to rareness, for example, participation in high‐risk sports and ischial spine sign, the highest sensitivities for detecting intraoperatively‐confirmed microinstability were obtained for female gender (93%) and Beighton score ≥2 (85%; Table 5). Beighton score ≥2 also showed a high specificity of 82%, a positive likelihood ratio of 4.73 and negative likelihood ratio of 0.18. Coding the 245 ‘normal’ Beighton scores as 0 did not change specificity and sensitivity for the optimal threshold (≥2). Because the optimal threshold increased to ≥4 when these were coded as 3, sensitivity reduced to 75%, but specificity increased to 91%. The female gender had positive and negative likelihood ratios of 2.08 and 0.12, respectively. Hip rotation range of motion on the index hip showed moderate sensitivity and specificity, with sensitivity higher for internal than for external rotation (72% vs. 67%) and specificity higher for external than for internal rotation (71% vs. 68%).

**Table 5:** Sensitivity and specificity for detecting intraoperatively‐confirmed microinstability.

	*n* (%) available	Sensitivity (95% CI)	Specificity (95% CI)	Youden's *J*‐statistic^c^
Age < 38.5	622 (100)	0.73 (0.70–0.77)	0.45 (0.42–0.49)	0.19
Female gender	622 (100)	0.93 (0.92–0.95)	0.55 (0.51–0.59)	0.49
Risk sport participant^a^	37 (5.9)	0.63 (0.47–0.78)	0.76 (0.62–0.90)	0.38
High‐risk sport participant^b^	37 (5.9)	0.50 (0.34–0.66)	0.97 (0.96–0.98)	0.47
Log roll index	60 (9.6)	0.66 (0.54–0.78)	0.36 (0.24–0.29)	0.02
Log roll opposite	44 (7.1)	0.60 (0.46–0.74)	0.33 (0.19–0.47)	−0.07
Beighton ≥ 2	513 (82.5)	0.85 (0.82–0.89)	0.82 (0.79–0.85)	0.67
Beighton ≥ 4	513 (82.5)	0.71 (0.67–0.75)	0.93 (0.91–0.95)	0.64
Beighton ≥ 5	513 (82.5)	0.63 (0.59–0.68)	0.93 (0.90–0.95)	0.56
Beighton ≥ 6	513 (82.5)	0.48 (0.44–0.52)	0.95 (0.93–0.97)	0.43
Ext rotation index ≥ 55	265 (42.6)	0.68 (0.62–0.74)	0.71 (0.66–0.77)	0.39
Int rotation index ≥ 30	266 (42.8)	0.72 (0.67–0.77)	0.67 (0.61–0.73)	0.39
Ext + Int rotation index ≥ 80	263 (42.3)	0.79 (0.74–0.84)	0.68 (0.62–0.73)	0.46
Ext rotation opposite ≥ 60	258 (41.5)	0.70 (0.64–0.75)	0.63 (0.57–0.69)	0.33
Int rotation opposite ≥ 35	259 (41.6)	0.66 (0.60–0.72)	0.69 (0.63–0.74)	0.35
Ext + Int rotation opp ≥ 95	256 (41.2)	0.77 (0.72–0.82)	0.64 (0.58–0.70)	0.41
LCEA < 35	618 (99.4)	0.73 (0.70–0.77)	0.56 (0.52–0.60)	0.29
Tonnis angle ≥ 3	40 (6.4)	0.82 (0.70–0.94)	0.31 (0.17–0.45)	0.13
Crossover	530 (85.2)	0.04 (0.03–0.06)	0.90 (0.87–0.92)	−0.06
Ischial sign	622 (100)	0.01 (0.00–0.01)	1.00 (0.99–1.00)	0.00

Abbreviations: Ext, external; Int, internal; LCEA, lateral centre‐edge angle; Opp, opposite hip.

^a^ Any of the following identified as main sport(s): gymnastics, dance/ballet, martial arts, figure skating, tennis, soccer/football, softball/baseball, golf, skiing.

^b^ Any of the following identified as main sport(s): gymnastics (including cheerleading/acrobatics), dance/ballet.

^c^ Youden's *J*‐statistic = sensitivity + specificity – 1.

From 38 predictive algorithms, including combinations of the best diagnostic microinstability indices with and without gender, those with the highest sums of sensitivity and specificity (or with 1 subtracted as Youden's *J*‐statistic) are ranked (Table 6). The best three of these algorithms were both ‘Female gender AND Beighton score ≥2’, (sensitivity 82%, specificity 84%); ‘Female gender AND either Beighton score ≥2 OR LCEA < 35°’ (sensitivity 93%, specificity 69%); and ‘both Beighton score ≥2 AND LCEA < 35°’ (sensitivity 60%, specificity 93%), none of these attaining an overall Youden index greater than Beighton score ≥2 alone. Having ‘either Beighton score ≥2 OR LCEA <35°’ raised sensitivity to 98% but at the expense of specificity (43%; Algorithm 10 in Table 6).

**Table 6:** Diagnostic statistics for algorithms based on combinations of indices of intraoperatively‐confirmed microinstability.

Algorithm^a^	Sensitivity (95% CI)	Specificity (95% CI)	+ve LR^b^	−ve LR^c^	Youden's *J*‐statistic^d^
1	0.82 (0.79–0.86)	0.84 (0.81–0.87)	5.25	0.21	0.67
2	0.93 (0.91–0.95)	0.69 (0.02–0.65)	3.01	0.10	0.62
3	0.60 (0.56–0.65)	0.93 (0.91–0.95)	8.81	0.43	0.53
4	0.58 (0.54–0.62)	0.94 (0.92–0.96)	10.50	0.44	0.52
5	0.67 (0.64–0.71)	0.83 (0.80–0.86)	3.94	0.39	0.50
6	0.72 (0.68–0.76)	0.78 (0.74–0.81)	3.21	0.37	0.49
7	0.96 (0.93–1.00)	0.48 (0.39–0.57)	1.84	0.08	0.44
8	0.71 (0.66–0.77)	0.73 (0.67–0.78)	2.59	0.40	0.44
9	0.96 (0.93–1.00)	0.43 (0.34–0.51)	1.68	0.09	0.39
10	0.98 (0.97–0.99)	0.41 (0.37–0.45)	1.65	0.05	0.39
11	0.74 (0.68–0.79)	0.63 (0.57–0.69)	2.00	0.42	0.37
12	1.00 (1.00–1.00)	0.22 (0.19–0.26)	1.29	0.00	0.22
13	0.76 (0.71–0.81)	0.43 (0.37–0.48)	1.32	0.57	0.18
14	0.99 (0.98–1.00)	0.19 (0.13–0.24)	1.22	0.06	0.18

Abbreviations: CI, confidence interval; LR, likelihood ratio. 1 Female gender AND Beighton score ≥2.

2 Female gender AND (Beighton score ≥2 OR lateral centre‐edge angle (LCEA) < 35°).

3 Both Beighton score ≥2 AND LCEA < 35°.

4 Female gender AND Beighton score ≥2 AND LCEA < 35°.

5 Female gender AND LCEA < 35°.

6 (Female gender OR Beighton score ≥2) AND LCEA < 35°.

7 Female gender AND (Beighton score ≥2 OR Participation in gymnastics, dance or ballet) AND LCEA < 35°.

8 Female gender AND (Beighton score ≥2 OR External+internal rotation on index side ≥80° OR External+internal rotation on opposite side ≥95°) AND LCEA < 35°.

9 Either Beighton score ≥2 OR Participation in gymnastics, dance or ballet OR LCEA < 35°.

10 Either Beighton score ≥2 OR LCEA < 35°.

11 Either Beighton score ≥2, OR External + internal rotation on index side ≥80° OR External + internal rotation on opposite side ≥95° OR LCEA < 35°.

12 Either Female gender OR Beighton score ≥2 OR LCEA < 35°.

13 Either Female gender OR Beighton score ≥2, OR. External + internal rotation on index side ≥80°, OR. External + internal rotation on opposite side ≥95° OR LCEA < 35°.

14 Female gender OR Beighton score ≥2 OR Participation in gymnastics, dance or ballet OR LCEA < 35°.

^a^ Algorithms listed in order of Youden's *J*‐statistic = sensitivity + specificity – 1.

^b^ Positive likelihood ratio (+ve LR).

^c^ Negative likelihood ratio (−ve LR).

^d^ Youden's *J*‐statistic = sensitivity + specificity – 1.

## DISCUSSION

The main finding of the present study was that from a range of potential presurgical markers of intraoperatively diagnosed microinstability, the Beighton score, with a very low threshold for a positive test of ≥2, was the best overall indicator (sensitivity 85% and specificity 82%). The Beighton test is a quick, reliable assessment that can be undertaken in a clinic or by patient self‐report. Because of the low threshold for detection of microinstability, test administration accuracy is likely to be less important. We acknowledge some uncertainty in this threshold, since 17.5% Beighton scores were unavailable and 39.4% were labelled as ‘normal <4’ without an exact numerical value specified. However, even if all normal Beighton scores were assumed to be 3, though unlikely given the distribution of known normal scores, the optimal threshold for detecting microinstability remains quite low, at ≥4. In contrast, many previously suggested indicators of microinstability were not diagnostically useful in the context of hip arthroscopy.

Women comprised approximately half (56%) of all hip arthroscopy patients. Female gender (sensitivity 93% and specificity 55%) was only exceeded by the Beighton score as an individual indicator of intraoperatively diagnosed microinstability, with female hip arthroscopy patients more than twice as likely as males to have this diagnosis. Similarly, when combinations of indicators, including Beighton score, risk sport participation, hip range of motion and LCEA were analysed for diagnostic accuracy, none outperformed Beighton score ≥2 alone, and only four were better than female gender alone. The best six algorithms included combinations of Beighton score ≥2, LCEA < 35° and female gender. Requiring all three raised specificity to 94%, whereas having either the Beighton score ≥2 or LCEA < 35° raised sensitivity to 98%. The latter combination might be a useful algorithm to alert surgeons to investigate for in‐surgery indicators of microinstability and consider intervention, such as a capsular plication procedure. That is, patients with no hypermobility and good acetabular coverage (LCEA ≥ 35°) can be ‘ruled out’ as very unlikely to have microinstability.

Several specific physical examinations for microinstability have been previously reported in the literature, for example, dial test [8], log roll test [21], abduction‐hyperextension‐external rotation (AB‐HEER) test [19], prone instability test [13], hyperextension‐external rotation (HEER) test [21], pull‐out test [14] and lateral hip instability test [49]. A recent exploratory regression analysis of predictors of instrumented distraction force under anaesthesia prior to hip arthroscopy noted that lower LCEA, female sex and a positive HEER test were predictors of microinstability determined by distraction ease [11]. Curtis et al. in that study did not include the Beighton score in their predictive model, while HEER assessment was not performed in the present study. Furthermore, Curtis et al. used a linear regression model, which did not allow determination of sensitivity or specificity for a clinical diagnosis [11].

In an earlier study of 194 consecutive hip arthroscopy patients by the same group, Hoppe et al. [19] reported the HEER test to have 71% sensitivity and 85% specificity for detecting intraoperatively confirmed microinstability, compared to the higher 85% sensitivity and similar 82% specificity reported for Beighton score ≥2 in the present study. Incorporating any one of HEER, LCEA or gender into a predictive algorithm would likely improve sensitivity at the expense of specificity, compared with HEER alone. Thus, the resulting metrics might be comparable to the Beighton score, alone or in combination with LCEA, reported here. Of the three hip examinations assessed, Hoppe et al. found the AB‐HEER test to have the best overall sensitivity (81%) and specificity (89%) for microinstability [19]. Although specificity was higher than reported here for the Beighton score, the 95% confidence intervals in that smaller study (81%–98%) overlap with those reported here (79%–85%) and are comparable. While some other studies have reported on diagnostic accuracy for detecting microinstability or borderline dysplasia, these have not employed an intraoperatively‐determined reference [14, 26, 49].

The Beighton score was initially developed for screening large populations for joint hypermobility [5], and has subsequently been adopted as a clinical tool for identification of generalised joint hypermobility. It is a simple assessment that has been shown to be reliably self‐reported [32], making it appropriate for telehealth consultation. Though widely used, its validity as a diagnostic tool and appropriate threshold values have been questioned due to variation with age, gender and ethnicity and poor correlation with individual joint laxity [29, 39, 46]. Definitions of abnormally high laxity in the context of hip arthroscopy vary from >6 [7], through >5 [1, 24], to >4 [8, 44]. Here, we found that the optimal threshold for detecting microinstability was very low (only ≥2). This suggests that even mild joint laxity is a strong indicator of microinstability in patients presenting for hip arthroscopy. The positive (4.73) and negative (0.18) likelihood ratios for Beighton score ≥2 indicate that having even mild generalised hypermobility increases the likelihood of microinstability diagnosis almost five times, whereas having no hypermobility reduces this likelihood by over 80%. Previous Beighton score thresholds defined by consensus or clinical impression for detecting microinstability may emphasise high specificity, or a lack of false‐positive cases (ruling in), over sensitivity, thus accepting a high false‐negative rate.

A large range of hip rotation is a commonly suggested indicator of microinstability. In this study, the best single hip rotation indicator (Youden index 0.46) was the total range of hip rotation (internal + external rotation ≥ 80°) on the index hip (sensitivity 79%, specificity 68%), followed by total range ≥95° on the opposite side (Youden index 0.41; sensitivity 67%, specificity 75%). Optimal diagnostic range‐of‐rotation thresholds were 5° smaller for index compared to opposite hips, supporting the possibility of pain‐related restriction. However, contrary to our hypothesis that index hip pain‐related guarding and restriction might obscure positive tests, index hips showed similar or better sensitivity than opposite hips. Bilateral symptoms may have complicated this picture. A threshold of >60° for internal or external rotation has been previously proposed [24]. Here, we report optimal thresholds for detection of microinstability to be close to this for external rotation (≥55° and ≥60° for index and opposite hips, respectively), but substantially less extreme for internal rotation (≥30° and ≥35° for index and opposite hips, respectively). Internal and external rotation had similar diagnostic accuracy on both hips.

In the present study, LCEA < 35°, representing a slightly shallow to shallow acetabulum, was the optimal threshold for detection of microinstability, but with relatively modest sensitivity (73%) and poor specificity (56%). We excluded patients with frank dysplasia (LCEA < 18) because they generally have overt hip instability, are commonly managed with periacetabular osteotomy rather than hip arthroscopy and are sparsely represented in arthroscopic microinstability studies [15, 34, 41]. Application of osteotomy instead of arthroscopic plication remains controversial for patients with borderline dysplasia (LCEA 18° or 20°–25°) [12, 28], and also depends on other bony indices, such as acetabular roof angle and femoral and acetabular version [34]. Although patients with frank dysplasia were not included in this analysis, a shallower acetabular socket appears to contribute to hip instability [25].

An important limitation of this study is that some examinations were not performed across the entire cohort, resulting in missing data for many parameters, and potential for bias because some measures were available more often in patients with microinstability than in those without. Restricting analysis to later years (2018–2020) substantially increased Beighton score availability (83% of this group compared to 27% in primary arthroscopies undertaken between 2009 and 2020 [3]). Despite this, the optimal diagnostic accuracy threshold calculated via ROC analysis was the same, and diagnostic performance remained essentially unchanged (sensitivity 85% in both analyses, and specificity only slightly reduced from 88% to 82%). Our assumption of a central score of 1.5 for those patients whose clinical records simply noted a ‘normal’ Beighton score of <3 might have biased results, but reanalysing these data with these patients categorised at either extreme of the range did not substantially alter findings. Another limitation, which also arises from examinations conducted in a clinical rather than a research context, is greater subjectivity in many of the index and reference measurements undertaken. Gender in our dataset, for example, is self‐reported and we do not have a record of sex at birth or of the existence of nonbinary sex characteristics. Furthermore, a changing awareness of the condition by the surgeon may have altered assessment techniques, even over the brief time frame of this study. Finally, although a previous study showed poorer patient‐reported outcome measures (PROMS) in patients with borderline hip dysplasia compared to those with frank dysplasia [23], we did not include preoperative PROMS as possible indicators of microinstability in this study, as we have found no differences in these measures between those with and without microinstability [4].

The generalisability of diagnostic metrics depends on the patient population. In this study, 22% of primary hip arthroscopies were retrospectively identified as having microinstability, though this proportion was only 12% across the longer 2009–2020 time frame [3]. Because microinstability was relatively uncommon in this surgeon's practice, diagnostic parameters might vary in cohorts with different demographic or sporting characteristics, for example, elite athletes in risk sports or activities, such as football or dance. We also excluded patients with acute intraarticular fracture, dislocation or recent history of significant trauma, since determination of preexisting microinstability was not possible. Therefore, our diagnostic metrics are not generalisable to these groups.

Microinstability as a diagnosis distinct from hip instability, in the context of lax soft tissue of the capsule and surrounding structures and borderline dysplasia, with or without excessive anteversion, is controversial. Nonetheless, the phenomenon of painful hip joint translation, in the absence of frank dysplasia, is widely recognised in the hip arthroscopy literature. Many hip arthroscopy patients with microinstability take several years to receive an accurate diagnosis, often seeing a range of health professionals. Because imaging findings may be subtle or fall within normal ranges [48], a pragmatic diagnostic algorithm based on commonly assessed clinical parameters could facilitate earlier recognition of microinstability and earlier initiation of preliminary nonsurgical rehabilitation. Although consideration of the primary cause of microinstability may help determine optimal management [21], timely arthroscopic intervention, including capsular plication, can be an effective approach [4].

## CONCLUSION

This study shows that among all individual and combinations of preoperative indicators evaluated, a Beighton score ≥2, indicating a low degree of hypermobility, was the most accurate and useful diagnostic test for intraoperatively‐confirmed microinstability in primary hip arthroscopy patients. Suspicion of microinstability should be heightened in patients with a Beighton score ≥2, especially in those of female sex and whose LCEA is <35°, to minimise the risk of a missed diagnosis.

## AUTHOR CONTRIBUTIONS

All authors contributed to the study conception and design. Matthew J. Brick and Catherine J. Bacon contributed to funding acquisition. Matthew J. Brick, Catherine J. Bacon, Rowan P. Auchterlonie, Gen Lin Foo and Man Lu contributed to data acquisition. Catherine J. Bacon completed the data analysis and prepared the original draft. All authors contributed to review and editing.

## CONFLICT OF INTEREST STATEMENT

Matthew J. Brick has received a speaker honorarium from Stryker. Other authors declare they have no financial interests.

## ETHICS STATEMENT

Ethical approval for this study, as part of an ongoing observational study, was obtained from the New Zealand Health and Disabilities Ethics Committees (17/NTA/269).

## Data Availability

The data that support the findings of this study are available from the corresponding author upon reasonable request.
